# A130 RETROGRADE ENDOSCOPIC ULTRASOUND-GUIDED ENTERO-ENTEROSTOMY USING A LUMEN-APPOSING METAL STENT FOR THE MANAGEMENT OF A HIGH-OUTPUT ENTEROCUTANEOUS FISTULA AND ILEAL STRICTURE IN A COMPLEX SURGICAL ABDOMEN

**DOI:** 10.1093/jcag/gwad061.130

**Published:** 2024-02-14

**Authors:** S Gupta, S Gupta, K Pawlak, J De Rezende-Neto, G May, J Mosko, N Calo

**Affiliations:** Therapeutic Endoscopy, St Michael's Hospital, Toronto, ON, Canada; Therapeutic Endoscopy, St Michael's Hospital, Toronto, ON, Canada; Gastroenterology, St Michael's Hospital, Toronto, ON, Canada; Therapeutic Endoscopy, St Michael's Hospital, Toronto, ON, Canada; Therapeutic Endoscopy, St Michael's Hospital, Toronto, ON, Canada; Therapeutic Endoscopy, St Michael's Hospital, Toronto, ON, Canada; Therapeutic Endoscopy, St Michael's Hospital, Toronto, ON, Canada

## Abstract

**Background:**

A 26-year-old male sustained significant traumatic thoracoabdominal injuries following a gunshot. He underwent several laparotomies, small bowel resections, an extended left hemicolectomy with end-colostomy formation, and a flap to close the abdomen. He subsequently developed a high-output enterocutaneous fistula (ECF) and loss of colostomy output. CT imaging confirmed an ECF from the ileum to the anterior abdominal wall and a severe ileal stricture distal to the fistula.

**Aims:**

In the context of his complex surgical abdomen and proximity of the ECF to the abdominal flap, surgical re-intervention was deemed high-risk. He was placed on total parenteral nutrition and referred for endoscopic management.

**Methods:**

Under fluoroscopic guidance, we injected methylene blue & contrast dye from the skin side of the ECF. A dilated segment of small bowel was filled, with no downstream passage of contrast (Fig 1A). Retrograde ileoscopy was performed with an Olympus pediatric colonoscope via the patient’s colostomy. 90 cm from the ileocecal valve (ICV), we encountered a benign enteric stenosis that could not be traversed. Contrast was injected, with fluoroscopy revealing a 10 cm long tortuous stenosis (Fig 1B), extending to the previously filled loop of small bowel. Given the length and characterof the stricture, endoscopic balloon dilation and enteral stenting were technically infeasible.

**Results:**

We proceeded to retrograde endoscopic ultrasound (EUS)-guided entero-enterostomy creation. With the aid of a guidewire, and under endoscopic, fluoroscopic and endosonographic guidance, a linear echoendoscope was advanced into the ileum via the colostomy, cecum and ICV. 50 cm from the ICV, we identified an adjacent dilated loop of small bowel (Fig. 1C). Water was instilled through the ECF, with the endosonographic view demonstrating filling, thus indicating this location to be upstream from the ECF. A 19-gauge needle was punctured through, with subsequent aspiration of methylene blue (Fig. 1D). We then created an EUS-guided entero-enterostomy using a 15mm lumen-apposing metal stent (Hot-AXIOS; Boston Scientific, Massachusetts, USA; Fig. 1E). Passage of methylene blue & contrast through the stent confirmed accurate deployment (Fig. 1F&G). With both the ECF & stricture bypassed, the patient’s colostomy output returned and ECF output diminished.

**Conclusions:**

Electrocautery-enhanced lumen apposition with metal stenting is well established. It can facilitate the formation of an anchored anastomosis across non-adherent luminal structures in a single-step fashion. Herein, we have reported a novel application of this technique in the management of a complex post-surgical trauma patient with a high-output ECF and deep enteric stenosis.

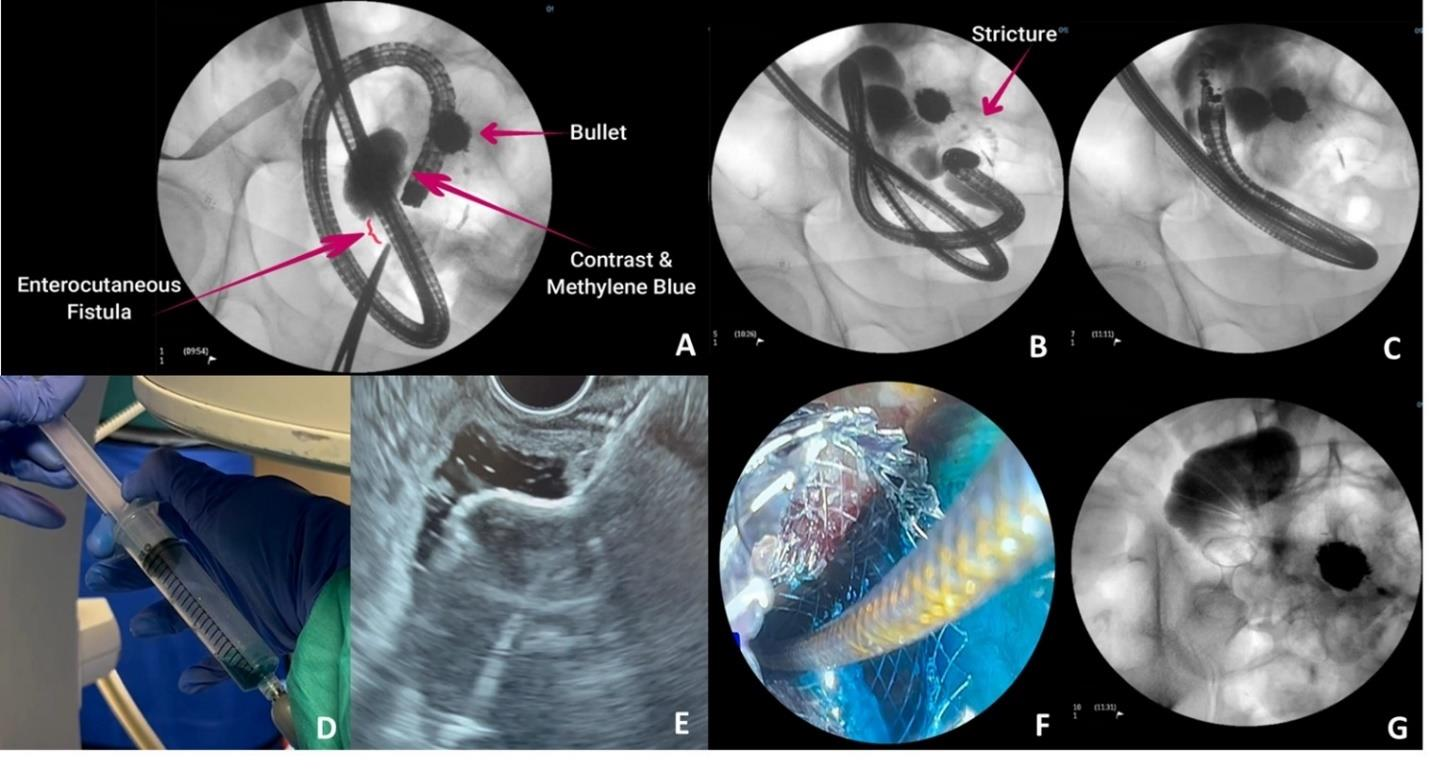

**Figure 1:** Retrograde EUS-guided entero-enterostomy

**Funding Agencies:**

None

